# High Hydrogen Yields During Continuous Photofermentative Valorization of Hydrothermal Carbonization Process Water by Engineered *Rhodobacter capsulatus*

**DOI:** 10.1111/1751-7915.70446

**Published:** 2026-11-06

**Authors:** Emma Barahona, Mario P. Díez, Ángel Valverde, Daniel Cuesta, Martina G. Vilaró, Viktor Cicimov, Amanda Prado, Juan A. Melero, M. Ángeles de la Rubia, Ángel F. Mohedano, Elena Diaz, Daniel Puyol

**Affiliations:** ^1^ Department of Biology, Higher School of Experimental Sciences and Technology University Rey Juan Carlos Madrid Spain; ^2^ Instituto de Investigación en Cambio Global, Higher School of Experimental Sciences and Technology University Rey Juan Carlos Madrid Spain; ^3^ Department of Chemical Engineering Universidad Autonoma de Madrid Madrid Spain; ^4^ Faculty of Technology and Metallurgy Ss. Cyril and Methodius University in Skopje Skopje North Macedonia; ^5^ Department of Chemical and Environmental Technology, Higher School of Experimental Sciences and Technology University Rey Juan Carlos Madrid Spain; ^6^ Instituto de Investigación de Tecnologías Para la Sostenibilidad, Higher School of Experimental Sciences and Technology University Rey Juan Carlos Madrid Spain; ^7^ Institute for Advanced Research in Chemistry Universidad Autonoma de Madrid Madrid Spain

**Keywords:** biohydrogen production, circular bioeconomy, nitrogenase‐driven hydrogen production, photofermentation, purple non‐sulphur bacteria

## Abstract

Photofermentative hydrogen (H_2_) production by purple non‐sulphur bacteria (PNSB) is a promising route for sustainable energy generation, although large‐scale application remains limited by substrate costs and process stability. Here, we demonstrate sustained H_2_ production using an H_2_‐overproducing *Rhodobacter capsulatus* Δ*hupAB* mutant cultivated on process water (PW) derived from the hydrothermal carbonization of food waste. Batch assays showed that buffered PW supports phototrophic growth, soluble chemical oxygen demand (COD) removal, and H_2_ production without requiring defined carbon sources. Based on these results, continuous photofermentation was operated at a hydraulic retention time of 3.5 days using diluted PW supplemented only with phosphate buffer, operating for more than 600 h, with sustained COD removal and H_2_ production. Average H_2_ yields reached 178 mL H_2_ g^−1^ COD during the stable operational period (100–350 h), comparing favourably with literature values. Carbon partitioning analyses revealed lower inorganic carbon formation under PW feeding, consistent with a redistribution of reducing equivalents towards nitrogenase activity. This behaviour was associated with lower biomass yields and enhanced H_2_ production. Minimal CO_2_ formation resulted in an almost pure H_2_ stream. These results demonstrate the feasibility of continuous photofermentative valorization of PW for coupling waste valorization with high‐purity biohydrogen production.

## Graphical abstract

Engineered *Rhodobacter capsulatus* enables nitrogenase‐driven hydrogen production from hydrothermal carbonization process water under continuous photofermentation, achieving high and sustained yields and supporting efficient waste valorization within a circular bioeconomy framework.

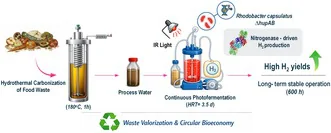

## 1 Introduction

Global energy still relies mainly on fossil fuels, whose depletion and greenhouse gas emissions highlight the urgent need for sustainable alternatives (Al‐Ghussain 2019; Karlilar Pata and Balcilar 2024). In this context, hydrogen (H_2_) has emerged as a key candidate, gaining increasing relevance both as an industrial commodity and as a strategic energy carrier for the transition to low‐carbon systems (Zhang et al. 2023). Owing to its high energy density (122 kJ g^−1^, about 2.7 times higher than gasoline), carbon‐free combustion, and fuel cell efficiencies of up to 50%, H_2_ is regarded as a particularly attractive option for decarbonizing hard‐to‐abate sectors such as transport and heavy industry (Nikolaidis and Poullikkas 2017). Despite its advantages, nearly 90% of global H_2_ is still produced from fossil fuels, yielding ‘grey hydrogen’ and associated CO_2_ emissions, which limit its role as a truly clean energy vector (Hossain Bhuiyan and Siddique 2025; Vezzoni 2024).

To overcome these limitations, biological hydrogen production (biohydrogen) offers a sustainable alternative (Zhang et al. 2024). This strategy is based on microbial processes such as dark fermentation and photofermentation, which transform organic substrates into H_2_ under mild and energy‐efficient conditions. Among them, photofermentation carried out by purple non‐sulphur bacteria (PNSB), such as *Rhodobacter capsulatus* , is particularly attractive. These microorganisms grow anaerobically in the presence of light and channel electron fluxes through nitrogenases to release H_2_ as a by‐product. PNSB are metabolically versatile, capable of utilizing a wide range of substrates while producing high‐purity H_2_ without the need for harsh operating conditions (Bayon‐Vicente et al. 2025; Capson‐Tojo et al. 2020; Ghosh et al. 2017; Hallenbeck and Ghosh 2009). However, many photofermentative systems still rely on defined or pure carbon sources, which may limit the economic feasibility of large‐scale applications.

One way to overcome these limitations is to use waste‐derived substrates, which, under the circular economy framework, can be transformed into valuable products such as biofuels while mitigating greenhouse gas emissions and waste disposal issues (Dahiya et al. 2018; Puyol et al. 2017). Food waste is particularly relevant in this context: in 2022, approximately 1300 million tons were discarded globally, representing about 19% of food available to consumers, in addition to another 13% lost along supply chains before retail (Sewell 2024). Overall, food loss and waste account for 8%–10% of global greenhouse gas emissions, emphasizing the need for efficient valorization strategies that can simultaneously reduce pollution and recover energy and resources. Hydrothermal carbonization has emerged as a promising technology for the valorization of wet biomass such as food waste, as it operates at relatively low temperatures and does not require prior drying of the feedstock. In addition to producing a carbon‐rich solid known as hydrochar, hydrothermal carbonization generates a liquid fraction termed process water (PW), which is typically characterized by high chemical oxygen demand (COD) and significant nutrient load (Ipiales et al. 2021; Mannarino et al. 2022). Although PW represents an environmental challenge due to its high organic load, acidic nature and the presence of potentially inhibitory organic compounds requiring treatment before discharge, its composition also provides opportunities for integration into circular economy strategies, including nutrient recovery, wet oxidation, recirculation and anaerobic digestion (Diez et al. 2024; Villamil et al. 2018).

Photofermentation has been successfully applied to several waste streams rich in volatile fatty acids (VFAs) and reduced organic compounds, including effluents from fermentation processes, thermal hydrolysis streams and food industry wastewaters. However, the photofermentation of hydrothermal carbonization‐derived PW remains largely unexplored. It represents a promising strategy to simultaneously alleviate the environmental burden of this liquid fraction and generate renewable H_2_. Within this framework, *Rhodobacter capsulatus* stands out among PNSB as a particularly suitable candidate due to its metabolic versatility and ability to valorize waste‐derived carbon sources for both energy recovery and the synthesis of natural biobased compounds (Allegue et al. 2020). Under nitrogen‐limited conditions, this organism can allocate reducing equivalents towards nitrogenase activity, resulting in H_2_ production. This may enable the conversion of the organic load of PW into high‐purity H_2_ and support its valorization within a circular bioeconomy framework.

In this work, we hypothesize that the use of hydrothermal carbonization PW, combined with continuous cultivation and an H_2_‐overproducing Δ*hupAB* mutant of *R. capsulatus* , promotes a redistribution of metabolic fluxes that enhances H_2_ production. To test this, we implemented a combined strategy integrating three complementary approaches. First, we evaluated PW as a waste‐derived carbon source. Second, we applied continuous cultivation to enhance process stability and long‐term productivity. Finally, we employed a genetically improved strain lacking uptake hydrogenase (*ΔhupAB*), a modification known to prevent H_2_ reoxidation and increase net H_2_ yields, and previously shown to enable H_2_ overproduction (Barahona et al. 2022, 2016). Together, these approaches were designed to evaluate an integrated strategy for continuous photofermentative valorization of hydrothermal carbonization PW, combining substrate evaluation, strain engineering and continuous cultivation to assess biohydrogen production, carbon partitioning and process performance.

## 2 Materials and Methods

### 2.1 Bacterial Strains and Growth Conditions

Two *R. capsulatus* strains were used in this study: SB1003 (WT) and its derivative mutant Δ*hupAB*. SB1003 is a spontaneous rifampicin‐resistant (Rifᴿ) mutant derived from the wild‐type B10 strain (Cullen et al. 1997). The Δ*hupAB* strain is a targeted deletion mutant lacking the *hupA* and *hupB* genes, which encode the large and small subunits of the uptake hydrogenase, respectively (Barahona et al. 2016).

*R. capsulatus* strains were cultivated in either rich YPS medium (Wall et al. 1975) or minimal RCV medium (Weaver et al. 1975) under phototrophic (anaerobic) or chemotrophic (aerobic) conditions at 30°C. Standard RCV medium contained 30 mM DL‐malate and 10 mM (NH_4_)_2_SO_4_ as sole carbon and nitrogen sources, respectively. For nitrogenase derepression, ammonium was omitted (RCV^−^). When used for solid cultures, media were supplemented with 1.5% (w/v) agar. Agar plates were routinely inoculated by streaking cells from glycerol stocks stored at −80°C. Rifampicin (25 μg mL^−1^) was added when required to maintain selection of the spontaneous Rifᴿ background. Petri dishes were incubated at 30°C in anaerobic jars flushed with N_2_ gas to remove residual O_2_. Illumination was provided by infrared lamps (Philips PAR38 IR, 175 W, E27, Philips Lighting, Netherlands) positioned externally to the anaerobic jars, delivering an irradiance of approximately 20 W m^−2^ with emission in the near‐infrared region suitable for phototrophic growth of PNSB.

Precultures were routinely grown in 150‐mL capped vials containing 60 mL of YPS medium. Cultures were inoculated from biomass previously grown on YPS agar plates. Following inoculation, vials were sparged with N_2_ to remove air completely and establish anaerobic conditions. Incubation was performed at 30°C under continuous infrared illumination delivering approximately 20 W·m^−2^, with 4 lamps positioned 25 cm above the vials to ensure uniform irradiance.

### 2.2 Process Water Preparation

Process water corresponding with the liquid fraction resulting from the hydrothermal carbonization of mixed food waste composed primarily of fruits and vegetables, including tomato, lettuce, apple, orange, leek, cabbage and grapes. The food waste was homogenized using a commercial grinder (Sammic XM‐30; Gipuzkoa, Spain) to a final moisture content of 91.5% and stored at −20°C until use. Hydrothermal carbonization was carried out in a 2 L stainless steel reactor (Parr Instrument Company, model 4524; Illinois, USA), loaded with 700 mL of homogenized food waste and stirred at 200 rpm. The reactor was hermetically sealed and heated to 180°C at a rate of 4°C min^−1^, maintained at the target temperature for 1 h, and subsequently cooled using an internal water coil at 10°C min^−1^ (Diez et al. 2024).

The resulting slurry, containing hydrochar and PW, was first passed through a cloth filter, followed by vacuum filtration through a 0.45 μm glass microfiber filter. The filtered PW was sterilized by autoclaving at 121°C for 20 min to prevent microbial growth, then characterized for pH, conductivity, and COD, and stored at 4°C until further use as the sole carbon source in photofermentation assays.

### 2.3 Batch Photofermentation Assays

Batch cultures were performed in previously autoclaved 150‐mL capped vials containing 60 mL of medium (headspace volume: 90 mL), inoculated with *R. capsulatus* strains to an initial optical density at 600 nm (OD_600_) of 0.02. Precultures of the WT and the Δ*hupAB* mutant were grown in YPS medium to mid‐exponential phase (OD_600_ ≈0.8), harvested, washed twice with carbon‐ and nitrogen‐free RCV medium, and used for inoculation. Vials were sealed with butyl rubber stoppers and flushed with high‐purity N_2_ for 1 min to ensure anaerobic conditions. Incubation was carried out at 30°C under continuous infrared illumination (20 W·m^−2^) at a distance of 25 cm, with orbital shaking at 70 rpm for 94 h.

Five culture media were tested: (i) RCV^+^, consisting of the standard RCV medium containing DL‐malate (30 mM) as the carbon source and ammonium sulphate (10 mM) as the nitrogen source; (ii) RCV^−^, identical to RCV^+^ but lacking ammonium sulphate while retaining DL‐malate (30 mM); (iii) PW + RCV^−^, consisting of diluted process water supplemented with all mineral and vitamin components of the RCV medium, but without DL‐malate or ammonium sulphate; (iv) PW + PB, consisting of diluted process water supplemented exclusively with phosphate buffer (pH 6.8) as the sole additional component; and (v) PW, consisting of diluted process water alone. Process water was diluted with deionized water to obtain a COD of 2.8 g L^−1^, equivalent to the organic load of 30 mM DL‐malate, thereby allowing direct comparison between experimental conditions. For the PW + RCV^−^ and PW + PB conditions, the corresponding supplements were added directly to the diluted PW to prepare the respective cultivation media. The pH was initially adjusted to 6.8 only in PW condition, since no phosphate buffer was present. For each condition and strain, three technical replicates were prepared, and the experiment was independently repeated twice (biological replicates).

Measurements were performed directly in each vial throughout the 94 h incubation period. Headspace pressure was recorded every 24 h using a digital manometer, followed by gas sampling with gas‐tight syringes for H_2_ quantification. Gas production calculations were corrected for the initial headspace pressure. Liquid samples were withdrawn aseptically at the same time points for COD determination, OD_600_ measurements, full‐spectrum absorbance (350–900 nm), and for the analysis of total ammonium (NH_4_^+^) and phosphate (PO_4_^3−^) concentrations.

### 2.4 Continuous Photofermentation Assays

Continuous photofermentation experiments were carried out in a 3 L jacketed glass photobioreactor (Pobel, Spain) equipped with a water recirculation system connected to a thermostatic bath to maintain the temperature at 30°C. The reactor headplate included multiple ports for liquid and gas inlets/outlets and instrumentation. A H_2_‐ sensor (BCP‐H_2_, BlueSens, Germany), previously calibrated for 1 h with N_2_ gas, was installed directly into one of the reactor's headplate ports for continuous monitoring of headspace H_2_ accumulation. The reactor was continuously stirred at 75 rpm and illuminated with two near‐infrared LED lamps (CMVision CM‐IR130, peak emission at 850 nm) positioned 10 cm from the reactor wall, delivering an estimated surface irradiance of approximately 20 W m^−2^. These NIR LED lamps produce negligible heat, preventing additional thermal input to the jacketed reactor system.

The reactor was inoculated with an anaerobic preculture of the Δ*hupAB* strain adjusted to an initial OD_600_ of 0.02. Before inoculation, the reactor was filled with 2 L of sterile medium and purged with N_2_ gas for 1 h to establish anaerobic conditions. Two experimental conditions were tested: (i) RCV^−^ control, consisting of synthetic RCV medium lacking ammonium; and (ii) PW + phosphate buffer (PW + PB), consisting of diluted PW adjusted to 2.8 g·COD L^−1^ and supplemented with phosphate buffer (pH 6.8). These conditions were selected to match the COD levels used in the discontinuous cultures.

The photofermentation assay was divided into two operational phases: an initial batch phase for system start‐up and biological acclimation, followed by continuous operation. During the batch phase, the reactor was operated in closed mode for 96 h until maximum H_2_‐production was reached. Subsequently, continuous operation was initiated using a peristaltic pump (Miniplus, Gilson, France) set at a constant feed and withdrawal rate of 25 mL h^−1^, corresponding to a hydraulic retention time (HRT) of 3.5 days. Throughout the experiment, the outlet gas line was connected to a 5 L Tedlar bag to collect and quantify the total biogas volume. Biogas was continuously vented from the reactor headspace to the Tedlar bag. Gas composition in the reactor headspace was monitored continuously using the in‐line H_2_ sensor and verified daily by GC. Liquid samples were also collected every 24 h for analysis of soluble COD, OD_600_, full‐spectrum absorbance (350–900 nm), total nitrogen (TN), ammonium and phosphate concentration, total organic carbon (TOC) and inorganic carbon (IC), total and volatile suspended solids (TSS and VSS), and VFAs. All operations, including assembly, sterilization, inoculation and sampling, were performed under sterile conditions to avoid contamination.

### 2.5 Analytical Procedures

Biomass growth was monitored by measuring the optical density at 600 nm (OD_600_) using a UV–visible spectrophotometer (Cole‐Parmer, USA). Additionally, the accumulation of bacteriochlorophyll *a* (BChl *a*) was evaluated as an indicator of phototrophic growth. Whole‐cell absorbance spectra were recorded in the range of 450–950 nm using a VIS–IR spectrophotometer (V‐630, Jasco, Madrid, Spain). The presence of characteristic absorption peaks at 805 and 850 nm was used to confirm BChl *a* production under phototrophic conditions (Madigan and Jung 2009).

Process water, as well as daily liquid samples collected from batch cultures and the photobioreactor, was analysed for total solids (TS) and VSS using standard gravimetric methods 2540B and 2540E, respectively (APHA 2005). Soluble chemical oxygen demand (sCOD) was determined according to the colorimetric method 5220D (APHA 2005). Total chemical oxygen demand (tCOD) was quantified following the procedure described by Raposo et al. (2008). pH was measured using a digital pH meter (Metria, model M21, Sweden). Ammonium and phosphate concentrations were determined using a Smartchem 450 automated discrete analyser (AMS Alliance, Italy), based on photometric colorimetric methods. Dissolved TOC, IC and TN were measured using a TOC‐V CPH analyser (Shimadzu, Japan) equipped with a 680°C catalytic combustion furnace, non‐dispersive infrared (NDIR) detection for carbon and chemiluminescence detection for nitrogen. TOC was determined by the NPOC method after acidification and CO_2_ stripping. IC was measured via direct acidification and degassing, and TN was analysed by high‐temperature oxidation followed by NO detection via chemiluminescence.

Volatile fatty acids were quantified by gas chromatography using a Shimadzu GC‐2030 system equipped with a flame ionization detector (FID) and a Nukol capillary column, as previously described by Diez et al. (2024). Briefly, samples were acidified with phosphoric acid, supplemented with crotonic acid as an internal standard and analysed under the chromatographic conditions reported therein.

Carbohydrate content was determined following the phenol–sulphuric acid colorimetric method described by DuBois et al. (1956). Soluble protein content was estimated from total Kjeldahl nitrogen (TKN) (Villamil et al. 2018) measurements and converted to protein equivalents using a nitrogen‐to‐protein conversion factor of 6.25, as previously reported by Jiang et al. (2014).

Glucose and lactate concentrations were quantified by high‐performance liquid chromatography (HPLC) using a Shimadzu LC‐2050C system (Shimadzu, Kyoto, Japan) equipped with a refractive index detector (RID‐20A). Separation was achieved using a Supelcogel C‐610H column (300 mm × 7.8 mm i.d.; Supelco Inc., USA) with 0.005 M H_2_SO_4_ as the mobile phase at a flow rate of 0.8 mL min^−1^. Analyses were conducted under isocratic conditions at 40°C with a total run time of 75 min.

Mineral and metal concentrations were determined by inductively coupled plasma optical emission spectroscopy (ICP‐OES) using an Iris Intrepid II XDL spectrometer (Thermo Fisher Scientific, USA).

The composition of gases in the headspace of the photobioreactor was analysed by gas chromatography using an Agilent Technologies GC System (model 7820) equipped with a thermal conductivity detector (TCD) and a series of stainless steel‐packed columns: one Porapak Q column (3 ft., 1/8″, 2 mm, 80/100 mesh), a second Porapak Q column (6 ft, 1/8″, 2 mm, 80/100 mesh), and a MolSieve 5A column (6 ft., 1/8″, 2 mm, 60/80 mesh). Argon was used as the carrier gas at a constant flow rate of 5 mL min^−1^. The oven temperature was set at 45°C and the detector operated at 220°C. Gas samples were injected manually using an external Luer‐lock connection. In addition, headspace H_2_ concentration was continuously monitored using a BCP‐H_2_ sensor (BlueSens GmbH, Herten, Germany) based on thermal conductivity, specific for non‐flammable binary gas mixtures. Readings were taken every 10 s and recorded using BlueVis software (BlueSens GmbH).

To quantify the total amount of H_2_ produced, the outlet gas stream was collected in 5 L Tedlar sampling bags (SKC Inc., USA) connected to the gas outlet of the reactor. The accumulated biogas volume was measured manually at each sampling interval using a 50 mL graduated syringe, allowing total H_2_ production to be calculated from the gas volume and H_2_ concentration. Hydrogen volumes were normalized to standard temperature and pressure (STP; 0°C and 1 atm).

### 2.6 16S rRNA Gene Sequencing for Taxonomic Validation

To verify the taxonomic identity and purity of *R. capsulatus* in the final effluents, 16S rRNA gene sequencing was performed on samples collected from the photobioreactor at the end of the continuous photofermentation assays operated with either RCV^−^ medium or PW + PB. Five millilitres of culture effluent were harvested by centrifugation, and genomic DNA was extracted using the HigherPurity Soil DNA Isolation Kit (Canvax Biotech) following the manufacturer's instructions. The extracted DNA was submitted to Fisabio (Valencia, Spain) for full‐length 16S rRNA gene amplification and sequencing using the PacBio Sequel platform generating circular consensus sequencing (CCS) reads. Raw sequence data were processed using QIIME2. Quality filtering and processing of long reads were performed to remove low‐quality sequences and potential chimeras. Amplicon sequence variants (ASVs) were inferred from the high‐quality CCS reads. Taxonomic classification was performed using the SILVA reference database (release 138). To further confirm the taxonomic identity of the dominant ASVs, representative sequences were compared against the NCBI GenBank database using BLASTn.

### 2.7 Statistical Analysis

For the statistical analysis of the COD consumption and H_2_‐ production data, all values obtained from the batch assays were analysed separately for each sampling time and bacterial strain. Data were first checked for normality and homoscedasticity before performing a one‐way analysis of variance (ANOVA) to assess differences among culture conditions. When significant effects were detected (*p* < 0.05), post hoc pairwise comparisons were conducted using Tukey's Honest Significant Difference (HSD) test to identify specific differences between treatments. The analysis was performed independently for WT and the Δ*hupAB* mutant to evaluate the effect of the culture condition within each strain without directly comparing between strains. Results are reported as adjusted *p*‐values, with statistical significance indicated by asterisks (*p* < 0.05, *p <* 0.01, *p <* 0.001). All statistical analyses were carried out in R (version 4.4.2) using the stats, dplyr, and multcomp packages. Data are presented as mean ± standard deviation (SD).

## 3 Results

### 3.1 Physicochemical Characterization of Process Water and Suitability as a Growth Substrate

The PW obtained from the hydrothermal carbonization of food waste was characterized by a high organic load and a predominantly soluble composition (Table S1). The PW exhibited an acidic pH (3.9) and high COD values (60.7 g L^−1^), corresponding to the soluble fraction, as measurements were performed on pre‐filtered samples. Total and volatile solids reached 40.0 and 30.2 g L^−1^, respectively, reflecting the concentration of organic compounds generated during hydrothermal carbonization.

The soluble organic fraction was mainly composed of carbohydrates and proteins, with soluble carbohydrates reaching 34.0 g L^−1^ and soluble proteins 5.7 g L^−1^. Low‐molecular‐weight compounds such as glucose (9.8 mg L^−1^) and lactate (15.0 mg L^−1^) were detected, together with ammonium (56 mg L^−1^), indicating the presence of readily assimilable carbon and nitrogen sources for phototrophic bacteria. In addition, PW contained relevant concentrations of macro‐ and micronutrients, including potassium, phosphorus, magnesium, calcium and trace metals (Table S1), which may support microbial growth but also contribute to the chemical complexity of the substrate.

Overall, this composition confirms that PW constitutes a highly concentrated and chemically complex effluent, requiring dilution and pH control prior to biological treatment, but offering a substantial reservoir of organic carbon and nutrients suitable for photofermentative valorization.

### 3.2 Comparative Analysis of Batch Cultures Under Photoheterotrophic Conditions Using Synthetic and Process Water‐Based Media

Batch cultures of the WT and the Δ*hupAB* mutant were evaluated under photoheterotrophic conditions to compare synthetic RCV medium and PW‐based media for H_2_ production.

Growth patterns were highly similar between the WT and the Δ*hupAB* mutant across all tested conditions, as reflected by OD_600_ measurements (Figure 1A,B). The highest biomass accumulation was observed in RCV^+^ and RCV^−^ media, with minimal differences between them. In contrast, growth in PW + RCV^−^ and PW + PB was markedly reduced relative to RCV, while no detectable growth occurred in the PW‐only condition (Figure 1A,B). A similar trend was observed for BChl a accumulation (Figure S1), with the most pronounced absorption peaks at 805 and 850 nm in RCV^+^ and RCV^−^, reduced intensities in PW + RCV^−^ and PW + PB, and no detectable peaks in PW alone.

**FIGURE 1:**
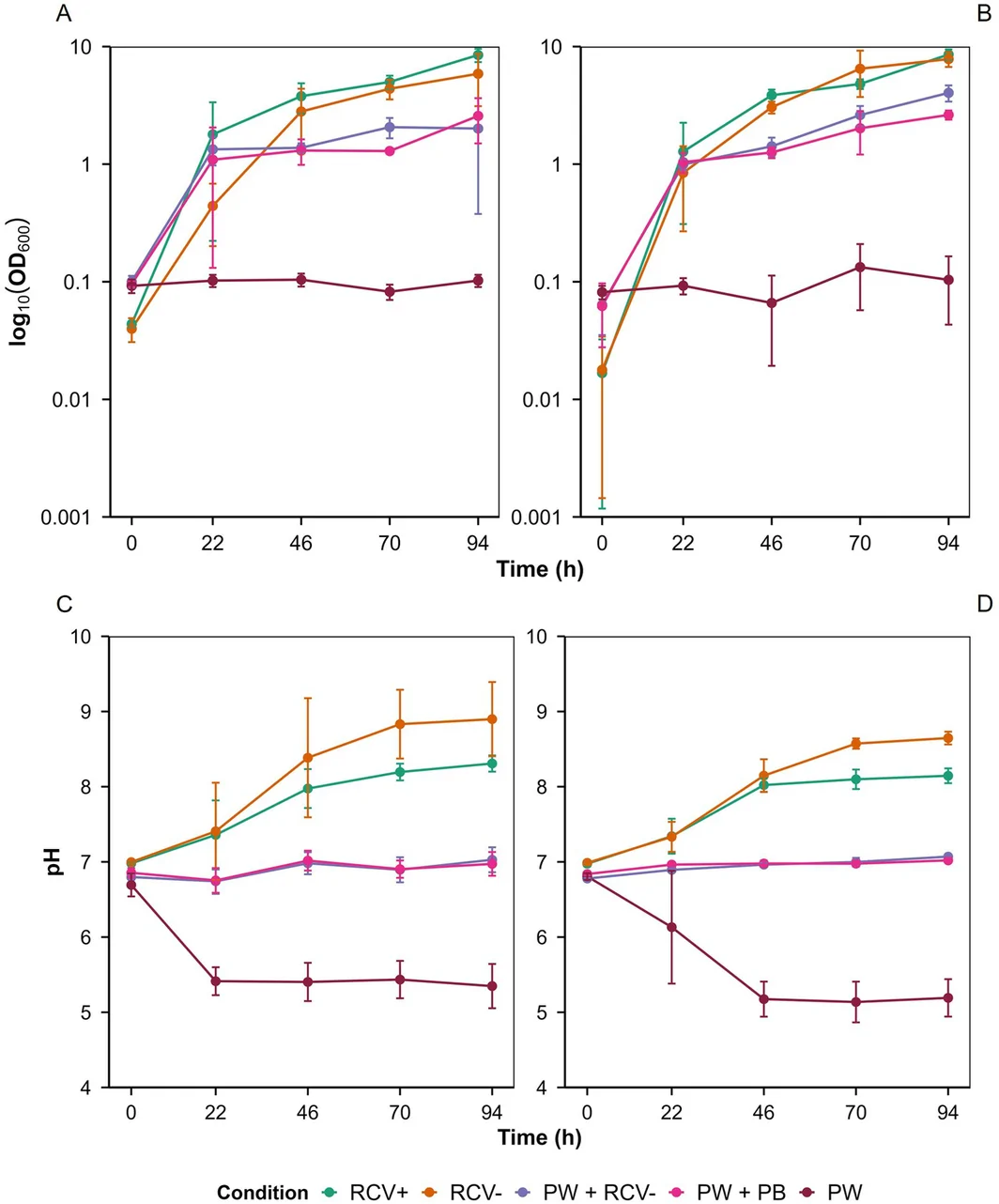
Growth and pH dynamics of *R. capsulatus* WT and Δ*hupAB* during batch photofermentation with synthetic and process‐water‐based media. (A, B) Growth curves (log_10_ OD_600_) of the WT (A) and Δ*hupAB* (B) strains cultivated for 94 h under photoheterotrophic conditions in different culture media: RCV^+^ (synthetic medium with malate and ammonium), RCV^−^ (without ammonium), PW + RCV^−^ (diluted process water supplemented with RCV medium lacking malate and ammonium), PW + PB (diluted process water with phosphate buffer, pH 6.8), and PW (process water alone, pH 6.8). (C, D) pH evolution for the WT (C) and Δ*hupAB* (D) strains during the same experiments. Data represent mean ± SD of two biological replicates, each comprising three technical replicates.

These observations were consistent with pH dynamics, which followed nearly identical trends for both strains (Figure 1C,D). In RCV^+^, pH increased from 6.8 to values between 8 and 9, likely due to malate assimilation, whereas a similar but less pronounced increase was observed under RCV^−^ conditions. In contrast, pH remained stable in PW + RCV^−^ and PW + PB, while in PW alone it decreased sharply within the first 48 h, reaching values close to 5 despite initial adjustment. In agreement with these trends, phosphate concentrations remained stable in all buffered conditions, confirming adequate buffering capacity, whereas PO_4_^3−^ levels in PW alone were negligible (Figure S2).

Soluble COD dynamics further supported these observations (Figure 2A,B). The initial COD in all conditions was approximately 3.0 g L^−1^, consistent with the target organic load set by malate in the standard RCV medium. Over the 94‐h incubation, COD decreased markedly in all conditions except PW‐containing treatments, reaching final values up to threefold lower than the initial concentration. In contrast, no COD removal was observed in the PW‐only condition for either strain. In the WT strain, COD removal was significantly higher in PW + RCV^−^ and PW + PB compared to RCV^+^ and RCV^−^ at 94 h (*p* < 0.001; Figure 2A). In the Δ*hupAB* mutant, PW‐based conditions also showed significantly higher COD removal than RCV conditions (*p* < 0.05; Figure 2B). No significant differences were observed between PW + RCV^−^ and PW + PB for either strain (*p* > 0.05). Overall, COD removal patterns were comparable between strains, although the WT showed slightly higher COD removal than the Δ*hupAB* mutant (*p* = 0.0099).

**FIGURE 2:**
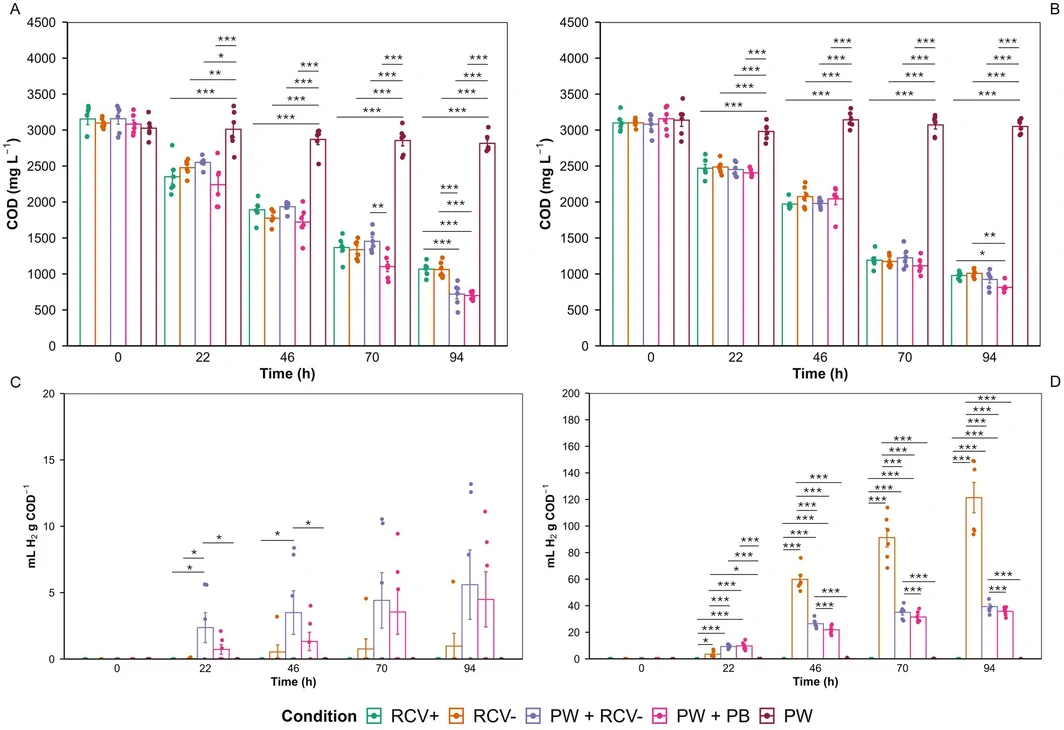
Soluble COD (sCOD) removal and hydrogen production in batch cultures of *R. capsulatus* WT and Δ*hupAB* under different culture conditions. (A, B) Soluble COD profiles for the WT (A) and Δ*hupAB* (B) strains cultivated under photoheterotrophic conditions for 94 h in RCV^+^, RCV^−^, PW + RCV^−^, PW + PB and PW media. (C, D) Hydrogen production (mL H_2_ g COD^−1^) over time for the WT (C) and Δ*hupAB* (D) strains grown under the same conditions. Bars represent mean values ± SD for two biological replicates with three technical replicates per condition. Statistical comparisons between conditions were performed independently for each sampling time within each strain using one‐way ANOVA followed by Tukey's post hoc test. Asterisks denote statistically significant differences: *P* < 0.05 (*), *p* < 0.01 (**), *p* < 0.001 (***). Note that panels C and D are displayed using different *y*‐axis scales to improve visualization of the lower H_2_ production by the WT strain.

Hydrogen production profiles (mL H_2_ gCOD^−1^) over the 94‐h batch incubation revealed clear differences between strains (Figure 2C,D). The WT strain showed consistently low H_2_ production across all conditions, with final values below 15 mL H_2_ gCOD^−1^. In contrast, the Δ*hupAB* mutant exhibited robust photofermentative H_2_ production, consistent with the absence of uptake hydrogenase. Under nitrogenase‐derepressing conditions (RCV^−^), H_2_ production reached peak values close to 150 mL H_2_ gCOD^−1^, with final values around 120 mL H_2_ gCOD^−1^ at 94 h. PW‐based conditions also supported substantial H_2_ production, although with a delayed onset compared to RCV^−^. This delay is consistent with the presence of residual ammonium detected in PW at 22 h (up to 25 ppm), which likely repressed nitrogenase activity. Consequently, the main phase of H_2_ production occurred after ammonium depletion, particularly between 22 and 46 h (Figure S3). Despite this initial delay, cultures grown in PW + RCV^−^ or PW + PB showed rapid H_2_ accumulation once ammonium was depleted. However, final H_2_ yields under these conditions were approximately 65% lower than in RCV^−^ at 94 h (Figure 2D). No significant differences were observed between PW + RCV^−^ and PW + PB (*p* > 0.05). As expected, no H_2_ production occurred when PW was used as the sole substrate.

### 3.3 Valorization of Process Water for Continuous Photobiological H_2_ Production

The experimental strategy followed a stepwise approach in which batch experiments were first used to identify the most suitable PW formulation and to compare the performance of the WT and Δ*hupAB* strains under PW‐based conditions. Based on these results, where PW + PB showed comparable H_2_ production, growth and substrate consumption to PW + RCV^−^, the more cost‐effective and simplified PW + PB formulation was selected for subsequent long‐term continuous photofermentative evaluation.

Continuous photofermentation experiments were conducted for more than 600 h. The operational strategy comprised three consecutive phases: (i) an initial batch phase to establish phototrophic growth before the onset of continuous feeding, (ii) a stable continuous operational period (approximately 100–350 h), which was used for quantitative comparison between cultivation conditions and (iii) a final operational phase (> 350 h), during which increased variability in biomass‐related parameters was observed. Unless otherwise stated, all quantitative analyses presented throughout this section refer to the stable continuous operational period.

Biomass dynamics during long‐term cultivation were monitored through OD_600_, Abs_805_, and VSS measurements (Figure 3A,B). In the RCV^−^ condition, OD_600_ and Abs_805_ increased during the batch phase, reaching maximum values close to 2.8 at 92 h, while VSS rose to nearly 4 g L^−1^. Upon transition to continuous cultivation, biomass remained relatively stable during the early phase (~100–350 h), with VSS averaging 4.98 ± 1.15 g L^−1^, consistent with fluctuations typically observed between 3 and 7 g L^−1^. During this period, OD_600_ remained stable at 2.00 ± 0.35, while Abs_805_ remained in a similar range (2.05 ± 0.51), indicating sustained phototrophic activity. From approximately 350 h onwards, a progressive decline in all biomass indicators was observed, with values decreasing steadily until ~375 h (Figure 3A).

**FIGURE 3:**
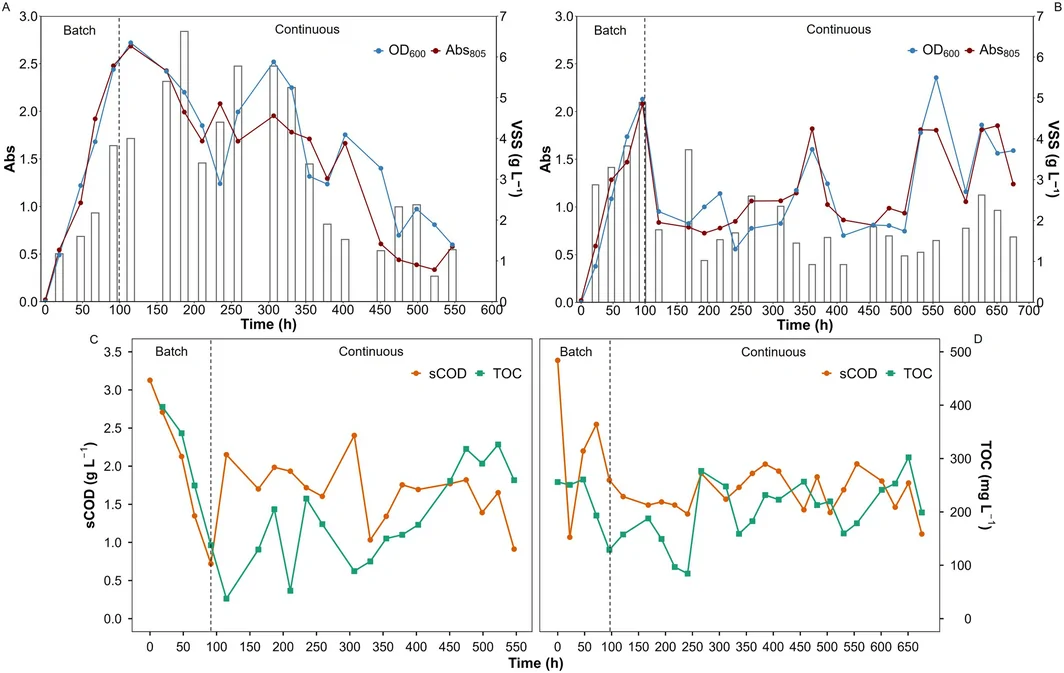
Biomass growth and carbon utilization profiles during continuous photofermentation of Δ*hupAB* mutant in synthetic and process‐water‐based media. (A, B) Time‐course profiles of biomass and pigment accumulation during batch start‐up and subsequent continuous operation. Optical density at 600 nm (OD_600_, blue), whole‐cell absorbance at 805 nm (Abs_805_, maroon), and volatile suspended solids (VSS, bars) are shown for cultures grown in RCV^−^ (A) and PW + PB (B) media. (C, D) Evolution of soluble chemical oxygen demand (sCOD, orange) and total organic carbon (TOC, green) under the same conditions. The vertical dashed line indicates the transition from batch to continuous mode (HRT = 3.5 days).

In the PW + PB condition, OD_600_ and Abs_805_ followed a similar initial trend during the batch phase, reaching maxima of 2.0–2.2 at 96 h, with VSS values around 4 g L^−1^. However, under continuous cultivation, biomass levels remained consistently lower than in RCV^−^, with OD_600_ averaging 1.00 ± 0.34, Abs_805_ averaging 0.98 ± 0.30, and VSS averaging 1.87 ± 0.88 g L^−1^ during the stable operational period (100–350 h). Compared with the RCV^−^ culture, average OD_600_, Abs_805_ and VSS values were approximately 2.0‐, 2.2‐ and 2.7‐fold lower, respectively (Figure 3B). Beyond ~350 h, transient increases in optical density were observed, reaching values up to ~2.3. In contrast, VSS remained comparatively stable during this late phase, suggesting that these fluctuations were primarily associated with changes in cellular pigmentation rather than sustained biomass growth (Figure 3B).

Parallel monitoring of physicochemical parameters revealed that pH progressively increased as the PB concentration was raised (from 1× to 2× in RCV^−^ and from 2× to 3× in PW + PB), demonstrating its direct role in maintaining culture stability (Figure S4). TN concentrations remained consistently higher in PW + PB than in RCV^−^, reflecting the presence of organic and inorganic nitrogen species in the PW and the reduced nitrogen assimilation under these conditions (Figure S5). This excess TN likely derives from soluble organic nitrogen compounds formed during the hydrothermal carbonization of food waste. Ammonium concentrations remained below the detection limit in both media throughout the experiments.

To further assess carbon availability and its implications for microbial metabolism, sCOD and TOC were analysed under both conditions (Figure 3C,D). During the initial batch phase, both media showed a rapid decrease in sCOD and TOC, reflecting active carbon assimilation by the cultures. During the subsequent stable phase (~100–350 h), the RCV^−^ condition maintained relatively consistent carbon levels, with average sCOD and TOC values of 1.76 ± 0.42 g L^−1^ and 130.32 ± 65.23 mg L^−1^, respectively (Figure 3C). In contrast, cultures grown in PW + PB exhibited greater variability in both sCOD and TOC, with average values of 1.62 ± 0.17 g L^−1^ and 171.36 ± 62.56 mg L^−1^, respectively (Figure 3D), indicative of a more heterogeneous carbon pool. This variability suggests the presence of multiple carbon fractions with differing bioavailability, likely supporting shifts in metabolic activity over time.

Notably, the relationship between sCOD and TOC in PW + PB deviated from the expected ratio for simple substrates, suggesting the presence of structurally diverse and partially oxidizable organic compounds. This pattern is consistent with the complex composition of the PW and may reflect differences in carbon bioavailability. Volatile fatty acids were analysed to characterize the dynamics of low‐molecular‐weight intermediates within the soluble organic fraction and to provide additional insight into metabolic fluctuations during continuous operation (Figure 4A,B).

**FIGURE 4:**
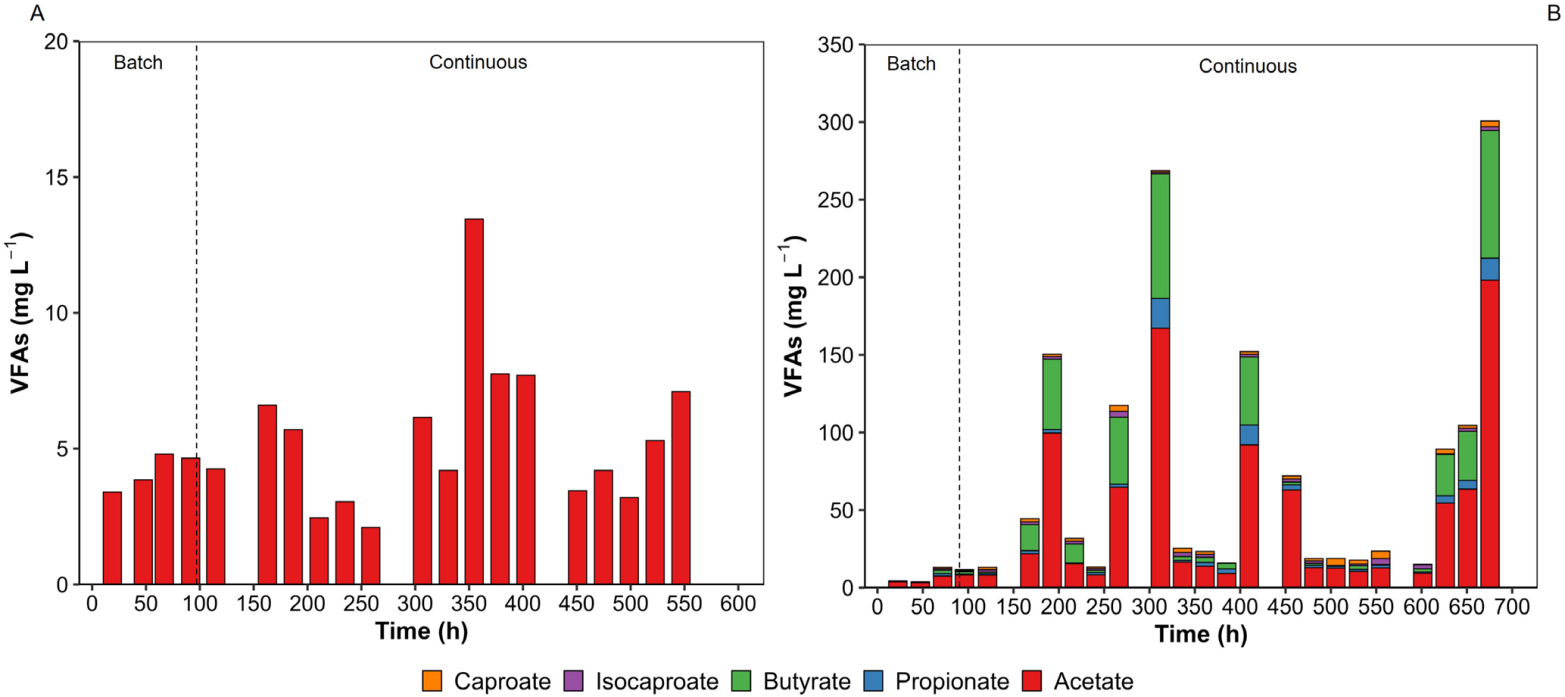
Volatile fatty acid (VFA) dynamics during continuous photofermentation of Δ*hupAB* in synthetic and process‐water‐based media. (A, B) Temporal profiles of individual VFAs measured during batch start‐up and subsequent continuous operation in RCV^−^ (A) and PW + PB (B) media. Concentrations of acetate (red), propionate (blue), butyrate (green), isocaproate (purple) and caproate (orange) are shown as stacked bars. The vertical dashed line indicates the transition from batch to continuous mode (HRT = 3.5 days). Note that panels A and B are displayed using different *y*‐axis scales to improve visualization of the lower VFA concentrations in the RCV^−^ medium.

As expected, no significant accumulation of VFAs was detected under the RCV^−^ condition, with only trace amounts of acetate observed in a few samples (Figure 4A), consistent with the direct assimilation of simple substrates under photoheterotrophic conditions. In contrast, under the PW + PB condition, a dynamic pattern of VFA consumption and transient accumulation was observed. The diluted PW initially contained 40–60 mg L^−1^ of VFAs, predominantly acetate (~95%), which was rapidly consumed during the early batch phase (Figure 4B). During the early continuous phase (~100–350 h), VFA concentrations remained generally low but highly variable, with an average total concentration of approximately 46 ± 55 mg L^−1^. Transient accumulation of acetate and butyrate was detected, suggesting intermittent imbalances between substrate uptake and metabolic processing, which coincided with decreases in OD_600_ (Figure 3B).

As operation progressed, VFAs were generally consumed, although sporadic accumulation peaks were observed, particularly around 300 h, which were associated with decreases in OD_600_ and increases in sCOD (Figure 4B). In the late phase (> 350 h), a progressive accumulation of VFAs, including propionate, was detected, reaching total concentrations of approximately 300 mg L^−1^. During this period, VFA concentrations increased slightly on average (48 ± 61 mg L^−1^) but remained highly variable, indicating a shift in metabolic balance. Overall, VFAs represented a minor fraction of the sCOD, accounting for approximately 10% during steady‐state operation, indicating that the majority of the carbon pool was composed of other organic compounds present in the PW. The late‐stage accumulation of VFAs is therefore consistent with a reduction in effective substrate assimilation capacity, likely reflecting a decline in metabolically active biomass or a transient imbalance between substrate inflow and microbial conversion rates.

Hydrogen production profiles revealed distinct behaviours between the two feeding regimes (Figure 5A). During the batch phase, H_2_ production was detected under both conditions but remained comparatively low, followed by a marked increase upon transition to continuous cultivation. To avoid bias from transient fluctuations, average H_2_ yields were calculated within the steady‐state operational window (100–350 h). Under these conditions, the RCV^−^ medium exhibited relatively stable H_2_ production, with an average yield of 26.14 ± 24.20 mL H_2_ g^−1^COD consumed. In contrast, the PW + PB condition showed substantially higher H_2_ production, with an average yield of 178.22 ± 188.60 mL H_2_ g^−1^ COD consumed during the same steady‐state period. A relatively high variability in H_2_ yields was observed under both cultivation conditions, although it was more pronounced in the PW + PB medium. The greater variability under PW feeding may be associated with the complex composition of the process water, which may promote intermittent shifts in substrate availability and metabolic activity. Despite this variability, H_2_ production was sustained throughout the stable operational period, demonstrating the feasibility of prolonged continuous photofermentative H_2_ production under PW feeding. Although transient peaks exceeding 600 mL H_2_ g^−1^COD were observed, these values represent short‐term metabolic fluctuations rather than steady‐state performance.

**FIGURE 5:**
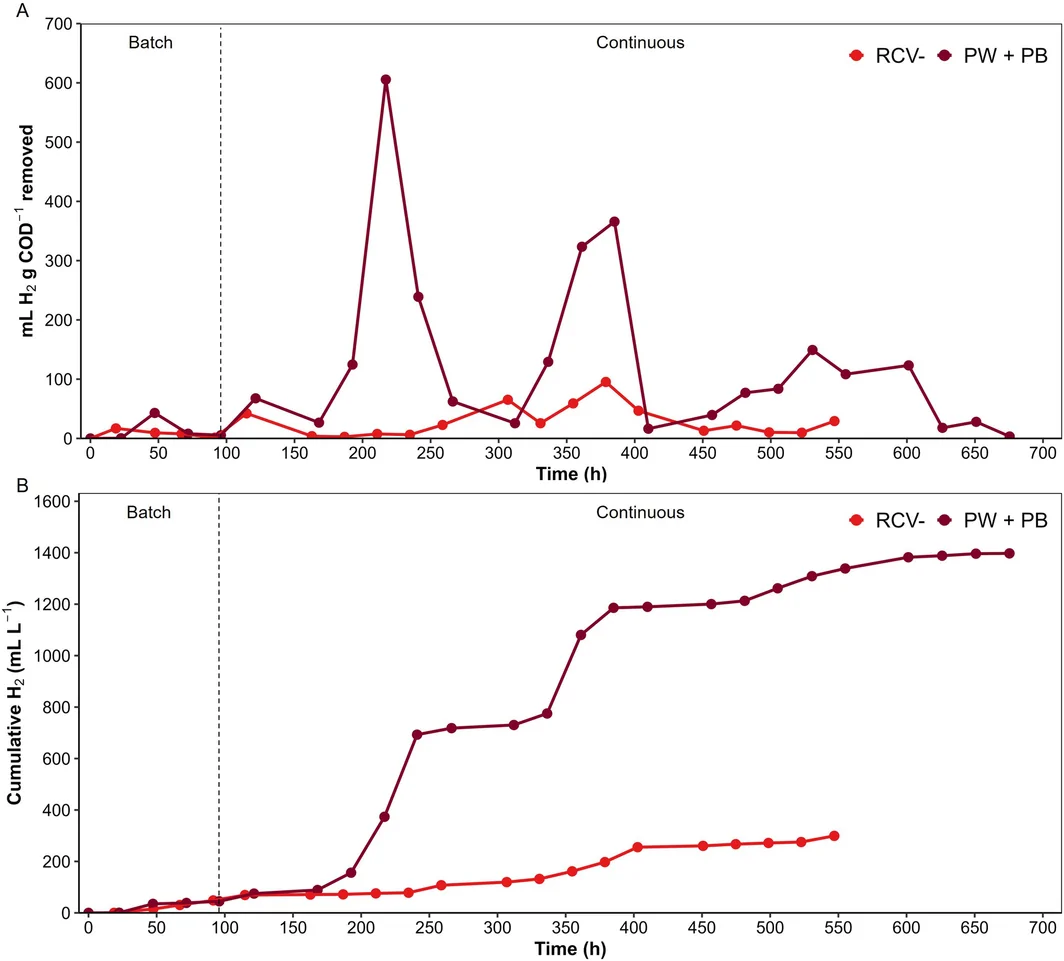
Hydrogen production profiles during continuous photofermentation of Δ*hupAB* mutant in synthetic and process‐water‐based media. (A) Time‐course of normalized H_2_ yields (mL H_2_ g COD^−1^ removed) during batch start‐up and continuous operation in RCV^−^ (red) and PW + PB (maroon) media. (B) Cumulative H_2_‐production (mL H_2_ L^−1^) under the same conditions. The vertical dashed line marks the transition from batch to continuous mode (HRT = 3.5 days).

Cumulative H_2_ production further emphasized the contrasting performance between feeding regimes (Figure 5B). In the RCV^−^ condition, total H_2_ accumulation remained limited, reaching less than 300 mL H_2_ L^−1^ after 550 h. In contrast, the PW + PB condition sustained prolonged H_2_ evolution, with stepwise increases that closely followed transient peaks in normalized yields. Production increased rapidly after the onset of continuous operation, surpassed 1000 mL H_2_ L^−1^ by 400 h and ultimately approached 1500 mL H_2_ L^−1^ after 650 h, highlighting the enhanced long‐term production capacity under PW‐based feeding (Figure 5B).

To confirm that long‐term H_2_ production was not associated with microbial contamination or community shifts, full‐length 16S rRNA gene sequencing was performed on samples collected at mid and late stages of operation for both conditions. All sequences (100%) were assigned to the genus *Rhodobacter*, with the dominant sequence showing 98.9% identity to *Rhodobacter capsulatus* SB1003, confirming the stability of the culture throughout the experiment.

## 4 Discussion

Photobiological H_2_ production by PNSB has attracted increasing attention as a sustainable route for renewable H_2_ generation. Among the strategies proposed to improve process performance, continuous cultivation and the use of waste‐derived substrates have emerged as particularly promising approaches for enhancing process stability while reducing production costs (Adessi et al. 2018; Feng et al. 2018; López Pérez et al. 2015; Savasturk et al. 2018; Zampieri et al. 2025).

This study demonstrates that hydrothermal carbonization PW can serve as an effective substrate for continuous photofermentative H_2_ production by the engineered Δ*hupAB* mutant of *R. capsulatus* . Compared with the synthetic RCV^−^ medium, PW is characterized by a high soluble organic load, acidic pH and a heterogeneous mixture of organic and inorganic compounds (Table S1), all of which are expected to impose physiological constraints on bacterial metabolism. Accordingly, while PW supported phototrophic growth and H_2_ production under batch conditions, its complex composition likely limited process performance. In contrast, these limitations were largely overcome during continuous operation, where continuous substrate supply and physiological acclimation enabled sustained and enhanced H_2_ production, reaching average yields of 178 mL H_2_ g^−1^ COD during the stable operational period. Together, these observations indicate that continuous cultivation enables the physiological adaptation required to efficiently valorize PW, thereby overcoming the limitations observed under batch conditions.

The batch experiments established the experimental basis for the subsequent continuous photofermentation assays by evaluating both the suitability of PW as a cultivation substrate and the performance of the H_2_‐overproducing Δ*hupAB* mutant of *R. capsulatus* (Barahona et al. 2016). Overall, the results demonstrate that, once buffered, PW provides a suitable environment for phototrophic growth and substrate utilization despite its greater chemical complexity compared with the synthetic RCV^−^ medium.

With respect to growth, no differences were observed between the WT and the Δ*hupAB* mutant under any of the tested conditions (Figure 1A,B), indicating that deletion of the uptake hydrogenase does not affect bacterial growth and confirming that the enhanced H_2_ production previously reported for the Δ*hupAB* mutant is not associated with altered biomass formation (Barahona et al. 2016). Both strains reached higher optical densities in RCV^+^ and RCV^−^, likely due to the rapid assimilation of malate, accompanied by an increase in pH above 8 (Figure 1C,D). In contrast, growth in PW + RCV^−^ and PW + PB was slightly lower, suggesting reduced assimilation efficiency of the more complex carbon sources present in PW (Figure 1A,B) (Diez et al. 2024; Ipiales et al. 2021), although pH remained stable due to buffering. Consistent with these observations, sCOD removal profiles were very similar between strains and across all buffered conditions (Figure 2A,B), indicating that a substantial fraction of the organic compounds present in PW remains bioavailable. Together, these findings demonstrate that the main limitation of PW is not substrate availability but the physicochemical constraints associated with its composition.

Hydrogen production, however, revealed a markedly different response. As expected, the WT strain produced only low H_2_ levels (< 15 mL H_2_ gCOD^−1^; Figure 2C), whereas the Δ*hupAB* mutant reached approximately 120 mL H_2_ gCOD^−1^ in RCV^−^ after 94 h (Figure 2D). Importantly, PW‐based media also supported relevant H_2_ production, reaching approximately 35–40 mL H_2_ gCOD^−1^ after 94 h. Although these values were significantly lower than those obtained in RCV^−^ (*p* < 0.001), no significant differences were observed between PW + RCV^−^ and PW + PB (*p* > 0.05) (Figure 2D). Considering the comparable substrate removal but lower biomass formation observed in PW (Figures 1B and 2B), these results suggest that part of the reducing equivalents generated during substrate assimilation may be redirected towards alternative metabolic processes, such as cellular maintenance or carbon storage, rather than being fully channelled to nitrogenase‐dependent H_2_ production (Capson‐Tojo et al. 2020; Hülsen et al. 2016; Puyol et al. 2017). This interpretation is consistent with the compositional complexity of PW, which contains a heterogeneous mixture of organic compounds that likely imposes additional metabolic demands (Ipiales et al. 2021; Tarhan et al. 2021).

One of the most relevant findings of the batch experiments was the absence of significant differences between PW + PB and PW + RCV^−^ in terms of H_2_ production (Figure 2D), demonstrating that additional supplementation with the synthetic RCV^−^ medium is unnecessary once pH is controlled. This observation indicates that PW itself provides the cofactors and nutrients required for Mo‐nitrogenase activity, including likely sources of molybdate. Furthermore, the ammonium concentration detected in PW (Figure S3) remained well below the threshold reported to repress Mo‐nitrogenase activity in *R. capsulatus* (~34 ppm) (Yakunin and Hallenbeck 1998), further supporting PW's suitability as a substrate for photofermentative H_2_ production. From a bioprocess perspective, these findings substantially simplify medium formulation without compromising photofermentative performance, thereby improving the process's practical applicability. Altogether, the batch experiments identified PW + PB as the simplest and most cost‐effective cultivation strategy capable of sustaining consistent H_2_ production, providing the rationale for the subsequent long‐term continuous photofermentation experiments. Continuous photofermentation of the Δ*hupAB* mutant was maintained for more than 600 h under both RCV^−^ and PW + PB conditions (Figure 3A,B). Optical density, bacteriochlorophyll *a* absorbance and VSS confirmed sustained biomass formation, although values were consistently lower in PW than in RCV^−^ (1.87 ± 0.88 vs. 4.98 ± 1.15 g VSS L^−1^ during the stable operational period), in agreement with batch assays and reflecting the higher complexity of the PW matrix. In addition, Abs_805_ closely followed OD_600_ trends under both conditions, supporting that optical measurements predominantly reflect active phototrophic biomass rather than non‐cellular suspended material. To further explore the relationship between biomass indicators, the OD_600_/VSS ratio was evaluated, revealing higher values in PW + PB than in RCV^−^ (0.75 ± 0.18 vs. 0.47 ± 0.11). This indicates that the relationship between optical density and suspended solids differs across conditions, suggesting that OD_600_ cannot be directly interpreted as a comparable proxy for biomass across media. These differences likely reflect variations in cellular pigmentation (e.g., bacteriochlorophyll content) and/or other optical properties under PW‐based cultivation.

Despite the markedly lower biomass concentration under PW feeding (1.87 ± 0.88 vs. 4.98 ± 1.15 g VSS L^−1^), overall carbon utilization remained largely unaffected. Batch experiments showed soluble COD removal reached approximately 66%–77% in all supplemented media (Figure 3C,D), demonstrating that replacement of the synthetic carbon source by PW did not impair carbon assimilation. Likewise, during continuous operation, both soluble COD and TOC removal remained comparable between PW + PB and RCV^−^, although TOC fluctuations were more pronounced under PW feeding, likely reflecting transient changes in soluble metabolic intermediates rather than incomplete substrate degradation. Average soluble COD removal was 43% ± 6% for PW + PB and 35% ± 15% for RCV^−^ despite the substantially lower biomass concentration observed under PW conditions. These observations indicate that the enhanced H_2_ production obtained with PW cannot be explained by greater substrate consumption, but rather by differences in carbon partitioning. This behaviour is consistent with previous studies showing that carbon removal in continuous purple phototrophic bacterial systems is strongly influenced by reactor configuration and biomass retention. While membrane‐based systems generally achieve higher substrate conversion efficiencies through improved biomass retention, conventional continuous stirred tank reactors may exhibit lower COD removal efficiencies due to biomass washout (Capson‐Tojo et al. 2020; Hülsen et al. 2016). Accordingly, the sustained removal of both sCOD and TOC achieved here demonstrates that stable carbon conversion can be maintained in a simple CSTR treating a chemically complex hydrothermal carbonization process water while maintaining H_2_ production over more than 600 h of continuous operation. Carbon partitioning analysis revealed clear differences between both media. Inorganic carbon accumulation remained consistently higher in RCV^−^ than in PW + PB (Figure S6), averaging 200.9 ± 62.7 and 108.8 ± 33.7 mg L^−1^, respectively, during the stable operational period (100–350 h). This indicates a greater extent of complete carbon oxidation in the synthetic medium. In contrast, the combination of lower IC formation, reduced biomass yields and enhanced H_2_ production observed under PW feeding is consistent with a mechanistic interpretation in which carbon and reducing equivalents are preferentially redirected towards nitrogenase‐dependent H_2_ production rather than biomass synthesis or complete carbon oxidation. The lower biomass yield observed under PW feeding is therefore consistent with a redistribution of assimilated carbon and reducing power towards H_2_ evolution rather than cellular growth. The metabolic behaviour observed under PW feeding is closely linked to its complex composition, dominated by carbohydrates and amino acids, while VFAs represent only a minor fraction of the initial COD (≈10%; Table S1). Under photoheterotrophic conditions, carbon assimilation by *R. capsulatus* is not governed by a purely glycolytic or fermentative scheme, but rather by the integration of central metabolism with photosynthetic energy conservation and redox balancing. In this context, reduced carbon substrates generate reducing equivalents that must be efficiently reoxidized to sustain cellular metabolism.

The transient accumulation of VFAs, such as acetate and butyrate during the initial stages of cultivation (Figure 4B), is therefore better explained by dynamic imbalances between substrate uptake, intracellular carbon processing and electron sink capacity, rather than by classical overflow metabolism. Their subsequent consumption under stable conditions is consistent with metabolic conditions supporting sustained nitrogenase activity and H_2_ production, whereas their accumulation reflects temporary metabolic constraints. Accordingly, VFA dynamics should be interpreted as indicators of intracellular redox balance rather than as primary drivers of H_2_ production.

Hydrogen production was markedly enhanced under prolonged continuous operation compared to batch conditions. In addition, the use of PW as substrate resulted in higher H_2_ yields than those obtained with the synthetic RCV^−^ medium, with average values of 178.22 ± 188.60 and 26.14 ± 24.20 mL H_2_ g^−1^ COD for PW and RCV^−^, respectively (Figure 5A). Maximum values above 600 mL H_2_ g^−1^ COD were observed during the stable operational period under PW feeding, although these peaks should be interpreted as transient metabolic fluctuations rather than representative steady‐state performance. To place these findings into context, representative literature values were normalized to mL H_2_ g^−1^ COD whenever possible and are summarized in Table 1. This comparison shows that, although higher H_2_ yields have been reported for defined substrates under optimized laboratory conditions, the continuous photofermentative valorization of real hydrothermal carbonization PW achieved in this study compares favourably with previously reported wastewater‐based systems. These findings further demonstrate that high H_2_ production can be sustained using a minimally supplemented waste‐derived substrate, highlighting the potential of this strategy for practical biohydrogen production.

**TABLE 1:** Comparison of representative H_2_ yields reported for selected biological H_2_ production systems under different cultivation conditions.

Study	Microorganism	Substrate	Operation	H_2_ yield (mL H_2_ g^−1^ COD)^a^	Remarks
Cai and Wang (2014)	*Rhodovulum sulfidophilum* TH‐79	Glucose	Batch photofermentation	~865	Pure substrate; value recalculated from reported glucose conversion
Uyar et al. (2009)	*Rhodobacter sphaeroides* O.U.001	Malate	Batch photofermentation	~700	Estimated from the reported 50% substrate conversion efficiency
Ozmihci and Kargi (2010)	*Rhodobacter sphaeroides* NRRL B‐1727	Dark fermentation effluent (mixed VFAs from wheat starch)	Continuous photofermentation	~185	Real fermentation effluent; highest yield obtained at HRT = 72 h.
Kim et al. (2012)	*Rhodobacter sphaeroides* KD131	Lactate	Continuous photofermentation	~480	Maximum H_2_ yield of 2.3 mol H_2_ mol^−1^ lactate at 20 mM lactate (38% substrate conversion efficiency); stable operation for > 250 days
Diez et al. (2024)	Mixed anaerobic culture	Hydrothermal carbonization PW	Continuous dark fermentation	20–40	Dark fermentation of the same substrate
This study	*R. capsulatus* Δ*hupAB*	Hydrothermal carbonization PW	Continuous photofermentation	178.2 ± 188.6 (max. > 600)	Minimal supplementation (phosphate buffer only); high‐purity H_2_

^a^ Hydrogen yields were normalized to mL H_2_ g^−1^ COD whenever possible to facilitate comparison among studies.

The relatively high variability in H_2_ yields observed under both RCV^−^ and PW + PB conditions suggests that temporal fluctuations are an inherent feature of prolonged continuous photofermentation. Such fluctuations likely reflect dynamic changes in nitrogenase activity and intracellular electron partitioning that occur even under stable operating conditions. The greater variability observed under PW feeding may additionally be attributed to the chemical heterogeneity of the PW, which can transiently influence substrate availability and intracellular redox balance. Despite these fluctuations, sustained H_2_ production throughout the stable operational period demonstrates the robustness of the process under continuous operation.

It should be noted that the continuous photofermentation experiments were conducted as single long‐term runs. Consequently, the results should be interpreted as demonstrating the feasibility of prolonged continuous photofermentative valorization of hydrothermal carbonization PW rather than providing a definitive assessment of process reproducibility. Nevertheless, the extended operational period (> 600 h), together with the consistent trends observed in biomass development, carbon utilization and H_2_‐production during the stable operational period, provides a robust basis for evaluating the behaviour of the system under continuous conditions.

Despite the generally higher yields reported for dark fermentation processes (Sanghvi et al. 2024), continuous photofermentation of PW using the Δ*hupAB* mutant clearly outperformed dark fermentation treating the same substrate. Reported values for dark fermentation of PW are typically in the range of 20–40 mL H_2_ g^−1^ COD (Table 1) (Diez et al. 2024), compared with an average H_2_ yield of 178.22 ± 188.60 mL H_2_ g^−1^ COD obtained during continuous photofermentation in this study. Overall, continuous photofermentation emerges as a more effective route for converting PW into H_2_ than dark fermentation, particularly when combined with targeted genetic modifications that enhance nitrogenase activity and H_2_ production.

Finally, gas composition analyses revealed very low CO_2_ production under both conditions, resulting in an almost pure H_2_ stream (Figure S7). This represents a major advantage over dark fermentation and other biological processes, where H_2_ is typically co‐produced with significant amounts of CO_2_ (Nath and Das 2009). Taken together, these findings support the potential of continuous photofermentation of PW using engineered PNSB to achieve high H_2_ yields while enabling the production of high‐purity biohydrogen, reinforcing its promise as a competitive strategy for waste valorization within sustainable energy frameworks.

Despite the promising performance demonstrated in this study, several challenges should be addressed before large‐scale implementation of continuous photofermentative PW valorization can be achieved. From a process engineering perspective, the use of PW supplemented only with phosphate buffer represents an important advantage, as it substantially simplifies medium formulation and has the potential to reduce operating costs compared with conventional synthetic media. However, the scalability of photobiological H_2_ production remains strongly dependent on photobioreactor design, particularly with respect to light distribution, energy requirements for illumination and the maintenance of homogeneous cultivation conditions under continuous operation. An additional challenge is the intrinsic variability of PW composition, which depends on feedstock characteristics and hydrothermal carbonization operating conditions. Besides biodegradable soluble organics, PW may contain phenolic compounds, furan derivatives, Maillard reaction products and other recalcitrant organics (Ipiales et al. 2021; Tarhan et al. 2021), which could interfere with photosynthetic metabolism or nitrogenase activity. Although stable operation for more than 600 h suggests that the PW used in this study did not exert major inhibitory effects under the selected conditions, future studies should evaluate process robustness across different PW compositions and identify potential inhibitory thresholds under continuous operation. Addressing these engineering and physiological challenges will be essential for translating the laboratory‐scale feasibility demonstrated here into robust industrial photofermentative biorefineries.

## 5 Conclusions

This study demonstrates the feasibility of continuously valorizing hydrothermal carbonization PW derived from food waste using an engineered *R. capsulatus* Δ*hupAB* strain. Batch experiments identified phosphate‐buffered PW as the simplest and most cost‐effective cultivation strategy, while prolonged continuous operation enabled sustained H_2_ production, effective COD removal and H_2_ yields that compare favourably with those reported for both photofermentation and dark fermentation of the same substrate. Importantly, the evolved gas contained only trace amounts of CO_2_, resulting in an almost pure H_2_ stream. These findings demonstrate that the combination of continuous cultivation and targeted genetic strategies unlocks the H_2_‐producing potential of complex waste‐derived substrates. Together, the biomass, carbon partitioning and H_2_ production data support a metabolic redistribution of carbon and reducing equivalents towards nitrogenase‐dependent H_2_ production. Overall, this work establishes an integrated strategy for sustainable photofermentative waste valorization and provides a strong foundation for the future development of high‐purity biohydrogen photobiorefineries.

## Author Contributions

**Emma Barahona:** conceptualization, investigation, funding acquisition, writing – original draft, methodology, software, data curation, supervision, formal analysis, visualization, validation, writing – review and editing, resources. **Mario P. Díez:** investigation, data curation, formal analysis, methodology, writing – review and editing. **Ángel Valverde:** investigation, data curation, formal analysis. **Daniel Cuesta:** investigation, formal analysis, data curation. **Martina G. Vilaró:** investigation, formal analysis, data curation. **Viktor Cicimov:** investigation. **Amanda Prado:** investigation, methodology, writing – review and editing, formal analysis. **Juan A. Melero:** project administration, resources, writing – review and editing, funding acquisition. **M. Ángeles de la Rubia:** supervision, funding acquisition, writing – review and editing. **Ángel F. Mohedano:** funding acquisition, writing – review and editing, project administration, resources. **Elena Diaz:** project administration, supervision, resources, funding acquisition, writing – review and editing. **Daniel Puyol:** methodology, supervision, funding acquisition, writing – review and editing, formal analysis.

## Funding

This work was supported by the BIVALIA‐CM project (TEC‐2024/BIO‐177), funded by the Comunidad de Madrid under the ‘Redes de Tecnología de la Comunidad de Madrid’ Program; the BioCat‐H2 project (SE180050116), funded by Fundación Iberdrola España; and the PID2022‐138632OB‐I00 project funded by MCIN/AEI/10.13039/501100011033. COST Action CA21146 (PURPLEGAIN), supported by COST (European Cooperation in Science and Technology).

## Conflicts of Interest

The authors declare no conflicts of interest.

## Supporting information


**Figure S1:** Whole‐cell absorption spectra of *R. capsulatus* WT and Δ*hupAB* grown under different culture conditions in batch mode. Panels A–E correspond to the WT strain and panels F–J to the Δ*hupAB* mutant. Specifically, A and F: RCV^+^; B and G: RCV^−^; C and H: PW + RCV^−^; D and I: PW + PB; E and J: PW. Spectra were recorded between 450 and 950 nm at several time points (0–94 h) during photoheterotrophic growth. The progressive darkening of purple tones indicates increasing cultivation time from 0 h (lightest) to 94 h (darkest). Characteristic absorption peaks near 590 nm correspond to carotenoids, whereas bands at approximately 805 nm and 850 nm are attributed to bacteriochlorophyll a (BChl a) light‐harvesting complexes LH2 and LH1, respectively.

**Figure S2:** Phosphate concentration profiles during batch photofermentation of *R. capsulatus* WT and Δ*hupAB* under different culture conditions. (A, B) Temporal evolution of phosphate concentration (mg PO_4_^3−^ L^−1^) during 94 h of incubation under photoheterotrophic conditions in RCV^+^, RCV^−^, PW + RCV^−^, PW + PB and PW media. Data for the WT strain are shown in (A), and for the Δ*hupAB* mutant in (B). Each point represents the mean ± SD of two biological replicates, each comprising three technical replicates.

**Figure S3:** Ammonium concentration profiles during batch photofermentation of *R. capsulatus* WT and Δ*hupAB* under different culture conditions. (A, B) Temporal evolution of ammonium concentration (mg NH_4_^+^ L^−1^) during 94 h of incubation under photoheterotrophic conditions in RCV^+^, RCV^−^, PW + RCV^−^, PW + PB and PW media. Data for the WT strain are shown in (A), and for the Δ*hupAB* mutant in (B). Each point represents the mean ± SD of two biological replicates, each comprising three technical replicates.

**Figure S4:** pH and phosphate dynamics during continuous photofermentation of Δ*hupAB* mutant in synthetic and process‐water‐based media. (A, B) Temporal evolution of pH (A) and phosphate concentration (mg PO_4_^3−^ L^−1^) (B) during batch start‐up and subsequent continuous operation under infrared illumination at 30°C in RCV^−^ (red) and PW + PB (maroon) media. The vertical dashed line marks the transition from batch to continuous mode (HRT = 3.5 days). In RCV^−^, phosphate buffer strength was increased from 1× in batch to 2× during continuous operation, whereas PW + PB cultures started at 2× and were raised to 3× in the continuous phase. Increasing buffer concentration correlated with higher and more stable pH values over time.

**Figure S5:** Total nitrogen (TN) concentration during batch start‐up and continuous photofermentation of Δ*hupAB* mutant in synthetic and process‐water‐based media. Temporal evolution of TN (mg L^−1^) under continuous illumination at 30°C in RCV^−^ (red) and PW + PB (maroon) media. The vertical dashed line marks the transition from batch to continuous operation (HRT = 3.5 days). TN values reflect the presence of both inorganic and organic nitrogen species.

**Figure S6:** Inorganic carbon (IC) concentration during batch start‐up and continuous photofermentation of Δ*hupAB* mutant in synthetic and process‐water‐based media. Temporal evolution of IC (mg L^−1^) under continuous illumination at 30°C in RCV^−^ (red) and PW + PB (maroon) media. The vertical dashed line marks the transition from batch to continuous operation (HRT = 3.5 days).

**Figure S7:** Composition of gases produced during continuous photofermentation of Δ*hupAB* mutant. (A) Synthetic medium (RCV^−^) and (B) process‐water‐based medium (PW + PB). The figure shows the relative composition of the biogas generated over time, including H_2_ (purple), CO_2_ (orange), and CH_4_ (grey). Percentages refer exclusively to the gases produced by microbial activity; the remaining gas fraction in the headspace corresponds to the nitrogen (N_2_) initially used for purging.

**Table S1:** Physicochemical characterization of process water obtained from the hydrothermal carbonization of food waste.

## Data Availability

All data generated or analysed during this study are included in this published article and its Figures [Link], [Link] and Table S1. Additional data are available from the corresponding author upon reasonable request.
