# Supplementary material for: High Hydrogen Yields During Continuous Photofermentative Valorization of Hydrothermal Carbonization Process Water by Engineered Rhodobacter capsulatus
Source: Microb Biotechnol. 2026 Nov 6;19(11):e70446. doi: 10.1111/1751-7915.70446 (PMC13742248; doi:10.1111/1751-7915.70446)
Supplement: Supplementary file 1 [file MBT2-19-e70446-s004.docx]

| **pH** | 3.9 |
| --- | --- |
| **TS** | 40.0 g L^-1^ ± 0.1 |
| **VS** | 30.2 g L^-1^± 0.8 |
| **tCOD** | 60.7 g L^-1^± 0.7 |
| **sCOD** | 60.7 g L^-1^± 0.7 |
| **NH_4_^+^** | 56 mg L^-1^± 0.2 |
| **Total proteins** | 5.7 g L^-1^± 0.1 |
| **Soluble proteins** | 5.7 g L^-1^± 0.1 |
| **Soluble carbohydrates** | 34.0 g L^-1^± 0.5 |
| **Glucose** | 9.8 g L^-1^± 0.1 |
| **Lactate** | 15.0 g L^-1^± 0.2 |
| **Calcium^*^** | 271.5 mg L^-1^± 2.1 |
| **Aluminum^*^** | 3.9 mg L^-1^± 0.1 |
| **Zinc^*^** | 1.7 mg L^-1^± 0.1 |
| **Manganese^*^** | 3.3 mg L^-1^± 0.1 |
| **Nickel^*^** | 0.1 mg L^-1^± 0.0 |
| **Potassium^*^** | 3009 mg L^-1^± 11 |
| **Sodium^*^** | 474.5 mg L^-1^± 5.5 |
| **Magnesium^*^** | 280.9 mg L^-1^± 2.2 |
| **Phosphorous^*^** | 270.7 mg L^-1^± 1.7 |

Table S1. Physicochemical characterization of process water obtained from the hydrothermal carbonization of food waste.

*All concentrations are expressed per liter of raw process water prior to dilution.*

**Inorganic elements determined by ICP-OES analysis.*
